# Assessing the challenges of e-learning in Malaysia during the pandemic of Covid-19 using the geo-spatial approach

**DOI:** 10.1038/s41598-022-22360-4

**Published:** 2022-10-15

**Authors:** Adi Jafar, Ramli Dollah, Nordin Sakke, Mohammad Tahir Mapa, Ang Kean Hua, Oliver Valentine Eboy, Eko Prayitno Joko, Diana Hassan, Chong Vun Hung

**Affiliations:** 1 Geography Programme, Faculty of Social Sciences and Humanities, Universiti Malaysia Sabah, 88400 Kota Kinabalu, Sabah Malaysia; 2 International Relations Programme, Faculty of Social Sciences and Humanities, Universiti Malaysia Sabah, 88400 Kota Kinabalu, Sabah Malaysia; 3 History Programme, Faculty of Social Sciences and Humanities, Universiti Malaysia Sabah, 88400 Kota Kinabalu, Sabah Malaysia; 4 Mathematic Programme, Faculty of Science and Natural Resources, Universiti Malaysia Sabah, 88400 Kota Kinabalu, Sabah Malaysia

**Keywords:** Diseases, Infectious diseases

## Abstract

The outbreak of the pandemic Covid-19 has transformed the education system in most countries worldwide. Following the lockdown measures in Malaysia, the Malaysian education system has fully transformed from conventional learning to online learning or known as e-learning as an alternative to minimize social contacts and physical communication to curb the transmission of Covid-19. In this regard, this study aims to identify the challenges faced by students in higher learning institutions throughout Malaysia during the implementation of the e-learning program. This study is based on a large sampling consisting of 2394 students from both public and private universities. The result from this study is analyzed through inferential methods such as the Spatial Analysis, the Principal Component Analysis, and the Mann–Whitney U test and through descriptive methods using the frequency analysis and the percentage analysis. Findings from this study suggest that location significantly influenced the challenges faced by students throughout the implementation of e-learning in higher learning institutions. For example, students in rural areas which can be identified as “vulnerable groups” are more likely to face both technical and connection with the internet access, tend to have a declining focus on learning and are prone to physical health problems, facing social isolation and low digital literacy compared to students in urban areas. Based on geographical analysis, students in Sabah, Perlis, and Melaka are most at risk of facing e-learning challenges. An anomaly case of students in Kuala Lumpur, however, posed another different result compared to other cities as they confront similar challenges with students in rural areas. This study provides the nuances of location and its implications for vulnerable groups that may put them at disadvantage in the e-learning program. Findings from this study will help to inform the relevant authorities and policymakers in improving the implementation of e-learning in Malaysia, especially towards the vulnerable groups so that it can be delivered more systematically and efficiently.

## Introduction

Educational achievement is one of the most important milestones in determining the quality of life. A low level of educational achievement is often associated with shorter life expectancy, poor health, and poor immunity during illnesses^1^. As such, the effectiveness of the education system must be emphasized to assist students’ achievement in education as this will affect the quality of their life. This includes reforms and improvements in educational policy and the delivery of teaching methods.

The outbreak of the pandemic Covid-19 which began in Wuhan, Hubei, China, in December 2019, has changed the educational system worldwide)^2–5^. The pandemic has shifted from conventional face-to-face teaching to distance learning, or known as e-learning, including education at the tertiary level^6,7^. The shift in the education system took place drastically in the wake of the current need worldwide to reduce social interaction to curb the transmission of the Covid-19^8^. However, this drastic change comes with hurdles as many developing countries are still new to the practice of e-learning^9^. Moreover, there are technological, facilities, and technical barriers that are prevalent in developing countries toward the implementation of e-learning. These include inadequacy of information and technology of communication (ICT), poor infrastructure facilities^9^, poor internet access^10^, deficit source of electricity^11^, poor living environment to learn at home^12^ and low digital literacy among teachers and students^13^.

Moreover, e-learning also poses several backdrops for the well-being of students. This includes mental health problems such as anxiety, depression, and stress^14^, physical health problems^15^, social isolation^12^, decreased sleep quality^16^ and negative impact on academic achievement^17^. However, geography and location also create a further disparity in the challenges of the e-learning system, particularly between rural and urban areas. For example, Zhu^18^ found that there is a digital divide between students in urban and rural areas in China. Almost half of the students in rural areas were unable to follow the e-learning program due to their limited ownership of electronic devices. Additionally, demographic factors have a significant impact in determining the amount of difficulty encountered by students. Cleofas and Rocha^19^ found, for instance, that the possession of computers (laptops and desktops) and low internet connection among college students with low socioeconomic position in the Philippines correlated to high levels of COVID-19-related anxiety.

Therefore, each of the problems and challenges in e-learning should be addressed accordingly using different mechanism empowerment strategies. However, before alternatives or resolutions are taken to minimize the barriers in e-learning, it is crucial to understand the nuances of problems that have been faced by students based on the consideration of their geographical location. This is to ensure the strategy and efforts to improve the e-learning system are more effective and systematic to mitigate the variety of challenges faced by students. Unfortunately, studies related to the challenges of e-learning from a geographical approach, particularly based on the mapping analysis and the differences in spatial aspects, are extremely rare and limited. The geographical approach which refers to the use of geospatial and mapping analysis is vitally important to demonstrate the varieties of barriers in e-learning, uniquely depending on the location, especially in developing countries.

Malaysia is considered a developing country that continues to implement e-learning, especially at the tertiary level^20^. Recent developments on Covid-19 show that the pandemic is an endless crisis and exacerbated by new variants such as the Delta, Omicron and Deltacron^21^. This inevitably became the main factor that led to the continuation of the e-learning system in Malaysia. However, so far, there has been no comprehensive study in the country that addresses the challenges of e-learning faced by students. This would be the main contribution of this study which aims to explore the challenges of e-learning through the utilization of geo-spatial and mapping analysis throughout all states, including the federal territories in Malaysia.

## Methods

### Study design

This study uses a quantitative approach. According to Jones et al.^22^, a survey is the most convenient method to accommodate a large sampling size. Therefore, due to the large sampling size, this study applies a cross-sectional survey to understand the challenges of e-learning among students in higher education in Malaysia. To eliminate social and physical interactions to reduce the risk of Covid-19, the data collection for this study has been conducted online using the KoBoToolbox application.

### Recruitment procedure

This cross-sectional survey was performed from 21 October 2021 to 6 December 2021. Respondents in this study are drawn from 9 private universities and 18 public universities throughout Malaysia (comprising 12 states and 2 federal territories). The sample size was determined using the purposive sampling technique with 2394 respondents. The minimum sample size to represent the total population exceeding 1,000,000 people or infinity with a 99% confidence level is capped at 463 people^23^. Therefore, the sample size for this study totaled 2394 respondents can be considered sufficient to represent the total population in Malaysia and the size has exceeded the required minimum sampling size. This study uses two popular social media platforms in Malaysia, WhatsApp and Facebook, to identify potential respondents.

### Study instrument

Some of the survey instrument questions of this study were emulated and modified from previous studies by Kim et al.^24^, Zembylas et al.^25^, and Adnan and Anwar^26^. These articles are written in English. The papers include a number of questions that are deemed appropriate for this study. Therefore, some of the questions in the articles (in English) were rewritten with a simpler format (in Malaysian) while retaining their original intent and meaning. The objective is to condense this study's e-questionnaire so that it is easier to understand, and respondents may spend less time answering questions. In general, this survey instrument contains two sections: the background information of the respondents (Part A) and the challenges faced by students in higher education during e-learning (Part B). Each question can be answered in the form of a Likert scale with five answer choices ranging from ‘1 (= strongly disagree) to ‘5 (= strongly agree). The total number of questions in Section B is 35 variables. Each of the questions in Section B is constructed in negative. This implies that a higher score indicates a greater degree of difficulty and vice versa. To verify the instrument's validity and reliability can be delivered and utilized in excellent condition, a pilot study with 50 respondents should be conducted beforehand^27,28^. The results of the analysis found that all variables (35 variables) were valid for use since the value of the correlation coefficient (r_xy_) is greater than the critical value for the Pearson’s Correlation coefficient r^29^ The minimum value of the correlation coefficient of this study is 0.299 exceeding the critical value for the Pearson’s Correlation coefficient r with a level of significance of 0.5%, which is 0.273^30^. The results of the reliability test also showed an alpha value of 0.935. This means that the research instrument for this study is suitable due to its alpha value in the excellent category^31^.

### Statistical analysis

The collection of raw data is analyzed using the IBM SPSS Version 26. To explain the respondents' background, this study utilized descriptive analyses such as frequencies and percentages. Apart from descriptive analysis, this study analyzed data using spatial analysis such as the Principal Component Analysis (PCA) and the Mann-Whitney U test.

**Figure 1:**
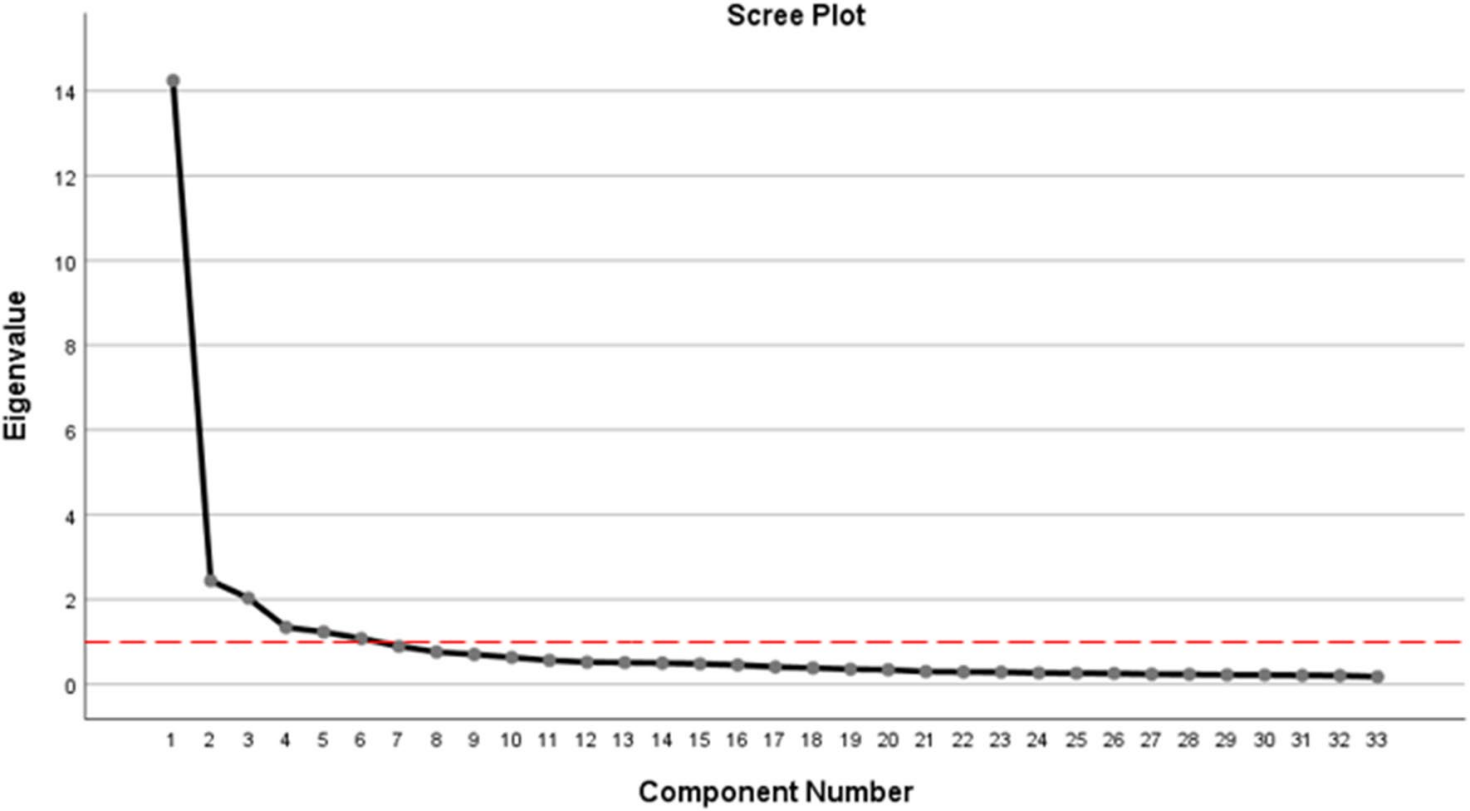
Component number.

The PCA functions as a data mining technique to summarize the 35 variables of "Construct B" into six components. The objective of the PCA analysis is to find and extract any essential information from the statistical data and to summarize this information as a collection of new orthogonal variables called principal components^32,33^. Since the value of the loading factor (commonality) of Variables B1 and B19 is less than 0.5, both variables had to be removed first before performing PCA analysis for the second time^34,35^. Bartlett’s test for the second time demonstrates that the PCA analysis showed a significant result (Χ^2^ = 54959.59, df = 528, p < 0.05), thus indicating that the sample of in this study was eligible for factorization. The number of components was determined with the help of Scree Plot, as shown in Fig. 1. Based on the figure, six components were produced with eigenvalues higher than 1^36^.

The cumulative value of variance from 6 components that were produced was 67.78 percent (Table 1). This means that 67.78% of the challenges faced by students in higher education in Malaysia are represented by these 6 components^37^. According to Williams et al.^38^, in the field of humanities, the variance described was as low as 50–60%. Subsequently, this examination's absolute variance rate should be acknowledged.

**Table 1:** Cumulative values of variance.

Component	Initial eigenvalues		
	Total	% Variance	Cumulative %
1	14.24	43.159	43.16
2	2.44	7.397	50.56
3	2.03	6.159	56.72
4	1.34	4.063	60.78
5	1.23	3.733	64.51
6	1.08	3.269	67.78
7–33	0.90–0.23	2.71–0.70	70.49–100

The results of the PCA analysis are introduced as a thematic map utilizing the Geography Information System (GIS), where the purpose is to demonstrate the dissemination of examples based on selected themes^39^. In order to produce a thematic map, the results of the PCA analysis will be merged with the spatial data. These spatial data were generated from open source which can be found at https://data.humdata.org/. This spatial data indicates the administrative boundaries of states in Malaysia. The State name, which is the primary key of the data, is then used to link these sets of data. The challenges of tertiary students were indicated on a choropleth map that depicts the allocation of values based on the recognition of color tones. A characteristic break then uses the order technique to progress the characterization by reducing the variation within classes and increasing the variance between each variant^40^.

Mann-Whitney U test, on the other hand, is to evaluate the differences in perception between students in urban areas and students in rural areas on the e-Learning system, where it is, by mathematical terms, elucidated as

1 $$U_{x} = n_{x} n_{y} + \left( {\frac{{n_{x} \left( {n_{x} + 1} \right)}}{2}} \right) - R_{x}$$

2 $$U_{y} = n_{x} n_{y} + \left( {\frac{{n_{y} \left( {n_{y} + 1} \right)}}{2}} \right) - R_{y}$$

where $n_{x}$ is the number of participants or observations in the first group, $n_{y}$ is the number of participants or observations in the second group, $R_{x}$ is the sum of the ranks assigned to the first group, $R_{y}$ is the sum of the ranks assigned to the second group.

### Ethical considerations

All methods were performed in accordance with the relevant guidelines and regulations. This study is conducted following the guidelines of the Ethics Committee set by the Universiti Malaysia Sabah (UMS) Review Board (Ref No UMS/FSSK6.2/100-2/2/3). All participants are supplied with an informed consent at the beginning of the online survey. In addition, this study provides written information, including its purpose and objectives in order for the respondent to understand before they participant in this study. There is a guarantee of privacy, anonymity, and confidentiality for all participants.

## Results

### Demographic characteristics

Of the total sample of this study, 1,723 (72%) were women, and only a total of 671 (28%) were men. Most of the respondents of this study also consisted of students who were married (2,339, 97.7%), Muslim (1,722, 71.9%) and pursued their studies at public universities (2,283, 95.4%). Further information regarding the demographic background of the respondents is shown in Table 2.

**Table 2:** Demographic characteristics of respondents (n = 2394).

Characteristics	Category	Frequency	Percent (%)
Gender	Male	671	28
	Female	1723	72
Marital status	Single	2339	97.7
	Married	55	2.3
Religion	Muslim	1722	71.9
	Christian	499	20.8
	Buddhist	116	4.8
	Hindus	41	1.7
	Others	16	0.7
Type of institution	Public university	2283	95.4
	Private university	111	4.6

### Challenges of e-learning

The results of the PCA analysis found that students in higher learning institutions faced six main challenges during the implementation of e-learning in Malaysia (Table 3). The main challenge of the e-learning can be seen with the aspect (Co1) of declining focus on learning (var (X) = 18.91%), followed by (Co2) the deterioration of physical health (var (X) = 12.62%) and (Co3) mental health disorders (var (X) = 11.41%). In addition, students also confronted issues with (Co4) technical and internet connection problems (var (X) = 10.37%) and (Co5) social isolation (var (X) = 7.36%). The last challenge is related to (Co6) which refers to students' inability to follow e-learning (var (X) = 7.11%).

**Table 3:** Analysis results of the main component extraction.

Components (domain)/item	Loading factor	Variance (%)
**Component 1 (Co1) decreased focus on learning**		
(B16) Lack of motivation as the learning environment at home is not similar to being at university	0.746	20.26
(B22) Easily bored due to limited knowledge on understanding the techniques in e-learning very limited e-learning learning techniques	0.735	
(B21) Difficult to focus on studies due to boredom on the e-learning teaching	0.734	
(B8) Lack of motivation as a result of lack of physical interaction with friends and lecturers	0.721	
(B18) Declining learning productivity	0.709	
(B17) Difficulty in understanding the content of the subject taught by lecturers	0.656	
(B23) Difficulty to concentrate on studies due to poor housing condition	0.646	
(B20) Difficulty in completing group assignments digitally	0.613	
(B9) Feeling alone	0.609	
(B15) Feeling drowsy during online classes	0.570	
(B24) Difficulty focusing due to disruption of other work at home	0.562	
**Component 2 (Co2) physical health problems**		
(B3) Neck pain	0.778	12.12
(B6) Eye fatigue	0.770	
(B4) Back shoulders pain	0.766	
(B2) Headaches	0.662	
(B5) Blurred vision	0.661	
(B7) Fatigue	0.578	
**Component 3 (Co3) problems with technical and internet connection**		
(B29) My internet access is limited due poor internet access in my home area	0.812	10.82
(B28) My internet access is limited due to due to expensive costs of internet access	0.806	
(B30) The prevalence of power outages in my housing area	0.738	
(B31) My laptop has a poor technical performance (slow capacity)	0.650	
(B32) Sharing learning devices, such as laptop, with my siblings	0.615	
**Component 4 (Co4) mental health problems**		
(B12) Easy to feel depressed (depression)	0.783	10.06
(B13) Easy to experience stress	0.772	
(B14) Easy to experience anxiety/restlessness (anxiety)	0.740	
(B10) Feeling isolated	0.547	
(B11) Lack of personal/physical attention	0.533	
**Component 5 (Co5) low digital literacy**		
(B35) Not easy to use e-learning as using other systems (traditional learning)	0.805	7.83
(B34) Not well-versed in e-learning	0.777	
(B33) I found that e-learning is difficult to use	0.697	
**Component 6 (Co6) social isolation**		
(B25) Not close with peers	0.791	7.41
(B27) Unable to recognize peers at university	0.754	
(B26) Difficulty communicating with through online communication	0.737	

### Comparison of e-learning challenges by state in Malaysia

**Figure 2:**
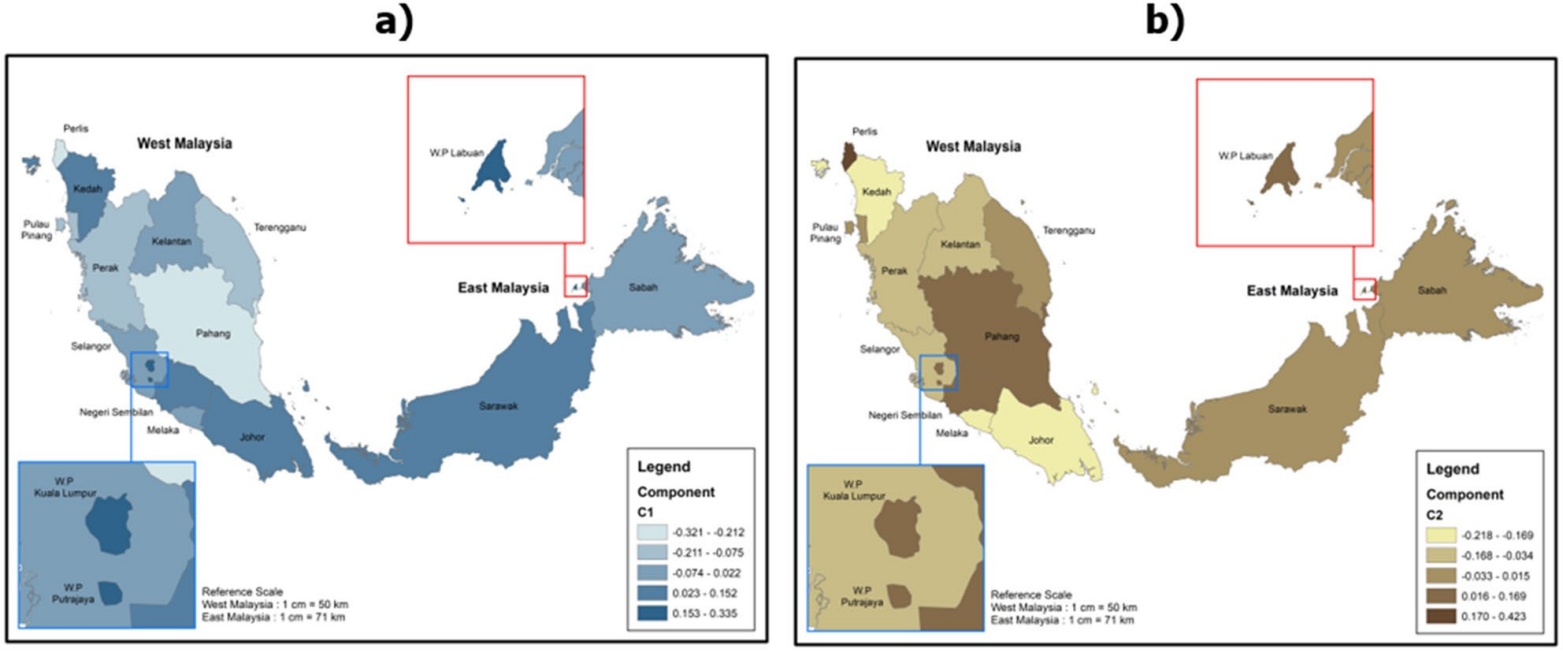
Challenges of e-learning from the aspect of the decreased focus on learning and physical health problems (the images were generated and modified from open source ‘Malaysia—Subnational Administrative Boundaries’ (https://data.humdata.org/dataset/cod-ab-mys) using ArcGIS Desktop 10.8.1 (https://www.esri.com/en-us/arcgis/products/arcgis-desktop/overview).

**Figure 3:**
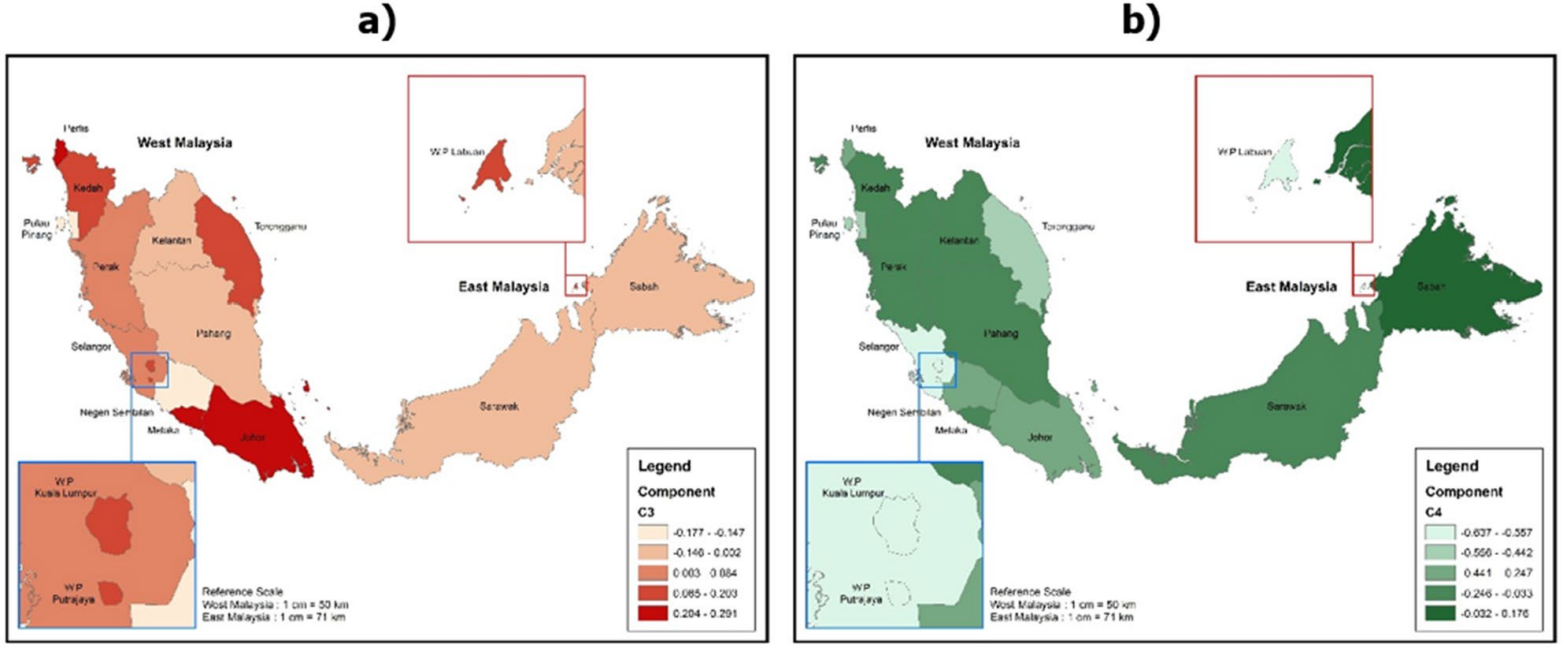
Challenges of e-learning from the aspect of mental health problems and, problems with technical and internet connection (the images were generated and modified from open source ‘Malaysia—Subnational Administrative Boundaries’ (https://data.humdata.org/dataset/cod-ab-mys) using ArcGIS Desktop 10.8.1 (https://www.esri.com/en-us/arcgis/products/arcgis-desktop/overview).

**Figure 4:**
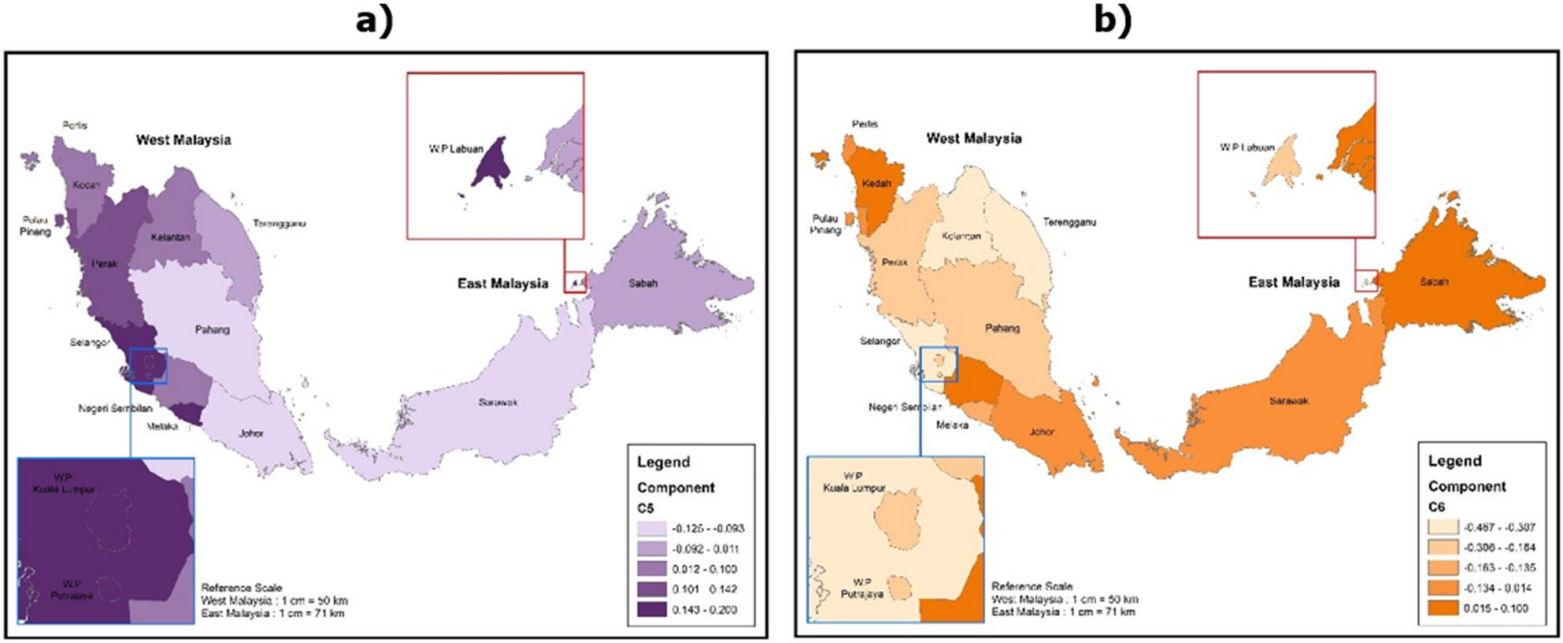
Challenges of e-learning from the aspect of social isolation and low digital literacy (the images were generated and modified from open source ‘Malaysia—Subnational Administrative Boundaries’ (https://data.humdata.org/dataset/cod-ab-mys) using ArcGIS Desktop 10.8.1 (https://www.esri.com/en-us/arcgis/products/arcgis-desktop/overview).

Based on the assessment of geographical considerations, each locality in Malaysia posed different challenges in e-learning. For example, students in Sabah are most likely to experience problems with technical and internet connection (Z-Scores = 0.1758) and low digital literacy (Z-Scores = 0.1000) (Figs. 3b, 4b). On the other hand, students in Kuala Lumpur enjoyed the most sophisticated technical and internet facilities for e-learning compared to students in other states in Malaysia (Z-Scores = -0.6373) (Fig. 3b). However, students in Kuala Lumpur are most likely to experience a decreased focus on learning (Z-Scores = 0.3347) and social isolation problems during e-learning (Z-Scores = 0.2000) (Figs. 2a, 4a). Students in the states of Perlis and Malacca are most likely to suffer from physical health (Z-Scores = 0.4226) and mental health (Z-Scores = 0.2913) problems (Figs. 2b, 3a).

### Differences in students' perceptions of e-learning challenges in urban and rural settings

The study also found significant differences concerning the challenges of the implementation of e-learning between students in urban areas and students in rural areas. This includes the aspects of decreased focus on learning (p ≤0.01), physical health problems (p = 0.001), problems with technical and internet connection (p ≤ 0.01), social isolation (p = 0.002) and low digital literacy (p ≤ 0.01). Compared to students in urban areas, students in rural areas are more likely to experience such problems (Co1, Co2, Co4, Co5 & Co6). However, for mental health problems, it was found that there was no significant difference (p = 0.297) between students in urban and rural areas as shown in Table 4.

**Table 4:** Results of the Mann–Whitney U test.

Component/domain	Location	Frequency (%)	Mean rank (MR)	*P* value
(Co1) Decreased focus on learning	Urban	988 (41.27)	1127.3	≤ 0.01
	Rural	1406 (58.73)	1246.8	
(Co2) Physical health problems	Urban	988 (41.27)	1142.7	0.001
	Rural	1406 (58.73)	1236.0	
(Co3) Mental health problems	Urban	988 (41.27)	1179.9	0.297
	Rural	1406 (58.73)	1209.8	
(Co4) Problems with technical and poor internet access	Urban	988 (41.27)	965.3	≤ 0.01
	Rural	1406 (58.73)	1360.6	
(Co5) Social isolation	Urban	988 (41.27)	1145.5	0.002
	Rural	1406 (58.73)	1234.1	
(Co6) Low digital literacy	Urban	988 (41.27)	1129.5	≤ 0.01
	Rural	1406 (58.73)	1245.3	

Mann–Whitney U (*p* value) at the level of significance (α = 0.05).

## Discussion

Students' geographical location at higher learning institutions has a crucial influence in determining their capacity to fully engage in the e-learning programme. Therefore, to ensure the effectiveness of the implementation of e-learning throughout all states in Malaysia, the government and policymakers should prioritize the overall improvements of e-learning, particularly for students in rural areas. Students in rural areas are more likely at a disadvantage given the variety of challenges they face due to poor basic infrastructure and facilities compared to students in urban areas. In addition, students in rural areas are more likely to experience challenges such as a deteriorating focus on studying, physical health issues, technical difficulties, a poor internet connection, social isolation, and a lack of digital competence in e-learning programme than students in urban areas (Refer to “Differences in students' perceptions of e-learning challenges in urban and rural settings” section). The results of this study are consistent with several previous research findings that students in rural areas are more commonly faced with technical issues and connection problems^11^ as well as low digital literacy^41^ during the implementation of the e-learning program.

A careful and detailed assessment of this study showed that students in Sabah scored the highest technical fault, poor internet access, and scored the lowest digital literacy (refer to Figs. 3b, 4b). This is not a surprise as Sabah is the poorest state in Malaysia^42^. Most of the districts and towns in Sabah are considered rural areas, and these areas are socially and economically backward in Malaysia. Sabah also scored the slowest economic growth rate compared to the 12 states in Malaysia^43^. Basic infrastructure facilities such as electricity supply, telecommunication network coverage, and roads in the state are still far behind compared to other states in Malaysia^44^. Therefore, it is not surprising that more than half (52%) of students in Sabah face difficulties during the e-learning program throughout the implementation of the national lockdown in 2020. Students in Sabah are commonly faced with a lack of internet access and the ownership of electronic devices (i.e., smartphones, computers, laptops)^45^. In turn, the low percentage of electronic device ownership among rural students will indirectly influence their improvement in digital literacy^46^.

University students with digital literacy and low internet access do not necessarily experience a decline in focus on learning. An anomaly was shown with students in Kuala Lumpur who are more likely to experience a decrease in focus on learning than students in Sabah (Fig. 2a). Besides that, university students in Kuala Lumpur also scored the highest social isolation compared to students in other states in Malaysia (Fig. 4a). At the same time, technical and internet facilities in Kuala Lumpur are considered the most sophisticated among all states in Malaysia (Fig. 3b). This implies that increasing internet access and digital literacy alone will neither boost academic accomplishment (increased learning focus) nor the degree of interaction amongst college students during e-learning. On the other hand, educators (lecturers, teachers, and tutors) should pay special attention to issues involving pedagogical elements that are critical in improving the quality and effectiveness of teaching and learning.

While the government could improve the implementation of e-learning, the improvisation of internet facilities and digital literacy is not enough to address the overall issues with e-learning. As shown in the case of Kuala Lumpur, even though students are equipped with good facilities and internet access, they are still vulnerable to other social issues such as isolation and decreased focus on learning.

Effective teaching strategies attract students to follow e-learning programs^47^. Therefore, in this pandemic era, educators need to be more creative, innovative and provide various teaching techniques to assist the focus and boost the interests of their students in the e-learning program. From a pedagogical point of view, the role of educators (lecturers, teachers, and tutors) should also be taken into account to improve the quality and effectiveness of their teaching. In addition, through the implementation of the e-learning program, the interaction between teachers and students must also be conducted two-way communications^48^. In other words, the teaching system must shift from the view of the learner as a sponge to the learner as an active construction of meaning^49^.

Two-way communication may assist in improving the psycho-emotional state as it would allow both individuals to engage openly^50^. This certainly would generate positive feelings of being validated through an interactive engagement between both individuals. In the context of e-learning, the two-way interaction between students and faculty members, including lecturers is limited. The barriers to two-way communication should be minimized, particularly through student relationships that are often defined through friendship. Social engagements among students themselves would certainly reduce the social isolation that university students in Malaysia commonly face. Educators should thus establish a teaching strategy and a comprehensive module to eliminate impediments to student communication and engagement. This would surely increase the students' interest in e-learning and improve their overall academic performance.

## Conclusion and recommendations

This study concluded that geographical location and residential environments impacted students' overall ability and effectiveness to participate in the e-learning program. Students in rural areas are more likely to face technical and connection problems, decreased focus on learning, physical health problems, social isolation, and low digital literacy than the urban students. Despite the distinctive case of students in Kuala Lumpur, the overall result from this study demonstrates that students in rural areas can be considered vulnerable groups facing more risks to various health and social problems as entailed in this study, especially in Sabah, Perlis, and Malacca. Therefore, priority should be given to the most vulnerable groups to ensure these students have the same privileges and opportunities in education as students in urban areas. Findings from this study are critical to inform the relevant policymakers and the government to improve the overall implementation of e-learning in Malaysia. This study provides the nuances of geographical location, especially towards the vulnerable groups, to inform the relevant policymakers and the government to improvise basic infrastructure and facilities so that e-learning can be conveniently accessible to all students in Malaysia. In addition, other pertinent social issues such as mental health and physical health throughout the implementation of e-learning also require an evaluation and intervention from the relevant policymakers to ensure the well-being of students is assured throughout the implementation of e-learning. This study also recommends that teaching professionals (lecturers, teachers, and tutors) who are actively engaged in the e-learning teaching process enhance their pedagogical skills. When e-learning is implemented, effective teaching strategies, in addition to technological factors, connection issues, and poor digital literacy, play a crucial role in enhancing academic achievement and minimizing social isolation among students. Moreover, this study contributes significantly to unveiling the varieties of e-learning challenges based on the intricacies of geo-spatial analysis and mapping which are currently limited in the literature on e-learning during the pandemic Covid-19. The novelty of this study will undoubtedly contribute to the existing literature on understanding the challenges of e-learning for better improvements in the education system in Malaysia.

## Supplementary Information

Supplementary Information 1.

## Data Availability

All data generated or analyzed during this study are included in this published article [and its supplementary information files].
